# Free Standing, Large-Area Silicon Nitride Membranes for High Toxin Clearance in Blood Surrogate for Small-Format Hemodialysis

**DOI:** 10.3390/membranes10060119

**Published:** 2020-06-06

**Authors:** Joshua J. Miller, Jared A. Carter, Kayli Hill, Jon-Paul S. DesOrmeaux, Robert N. Carter, Thomas R. Gaborski, James A. Roussie, James L. McGrath, Dean G. Johnson

**Affiliations:** 1 SiMPore, Inc. 150 Lucius Gordon Drive, Suite 110, West Henrietta, NY 14586, USA; jmiller@simpore.com (J.J.M.); jcarter@simpore.com (J.A.C.); jsd3691@gmail.com (J.-P.S.D.); roussie@simpore.com (J.A.R.); 2 Biomedical Engineering Department, University of Rochester, Rochester, NY 14627, USA; khill18@ur.rochester.edu (K.H.); jmcgrath@bme.rochester.edu (J.L.M.); 3 Mechanical Engineering Department, Rochester Institute of Technology, Rochester, NY 14623, USA; ncbme@rit.edu; 4 Biomedical Engineering Department, Rochester Institute of Technology, Rochester, NY 14623, USA; trgbme@rit.edu

**Keywords:** nanomembranes, model, hemodialysis

## Abstract

Developing highly-efficient membranes for toxin clearance in small-format hemodialysis presents a fabrication challenge. The miniaturization of fluidics and controls has been the focus of current work on hemodialysis (HD) devices. This approach has not addressed the membrane efficiency needed for toxin clearance in small-format hemodialysis devices. Dr. Willem Kolff built the first dialyzer in 1943 and many changes have been made to HD technology since then. However, conventional HD still uses large instruments with bulky dialysis cartridges made of ~2 m^2^ of 10 micron thick, tortuous-path membrane material. Portable, wearable, and implantable HD systems may improve clinical outcomes for patients with end-stage renal disease by increasing the frequency of dialysis. The ability of ultrathin silicon-based sheet membranes to clear toxins is tested along with an analytical model predicting long-term multi-pass experiments from single-pass clearance experiments. Advanced fabrication methods are introduced that produce a new type of nanoporous silicon nitride sheet membrane that features the pore sizes needed for middle-weight toxin removal. Benchtop clearance results with sheet membranes (~3 cm^2^) match a theoretical model and indicate that sheet membranes can reduce (by orders of magnitude) the amount of membrane material required for hemodialysis. This provides the performance needed for small-format hemodialysis.

## 1. Introduction

In 2017, the incidence of end-stage renal disease (ESRD) in the United States was over 124,000 and more than 61% of these ESRD patients used hemodialysis (HD) for renal replacement therapy [1]. However, ESRD patient mortality has remained nearly the same for twenty years, with no significant change (<1 year increase) for patients over the age of 50 [1]. This can be attributed to the dearth of improvements in hemodialysis technology over the last 40 years. A number of problems with the current therapies still persist; patients receiving thrice-weekly HD treatments report feeling fatigued and depressed, and their overall quality of life is generally poor. In addition, in-center thrice-weekly treatments have been linked with increased risk of cardiovascular events, emergency room visits, and mortality due to the extra-long interdialytic period [2,3,4]. Continuous hemodialysis will be able to improve patients’ quality of life by utilizing a small extracorporeal circuit containing a membrane capable of achieving prescribed toxin clearances in a wearable or implanted device. The extreme thinness (<100 nm) of these membranes combined with their high porosity will enable such a wearable or implantable format needed for continuous (intensive) HD treatments. In addition, the membranes, which have pores that can be tuned to separate molecules based on size, allow the diffusion of small molecules and exhibit good biocompatibility [5]. By incorporating silicon nitride nanomembranes into a benchtop hemodialysis system, we are able to show that they have a maximum theoretical clearance, *Ko*, of ~45,000 mL/min/m^2^. This is 30 times greater than that of traditional polymer-based membranes.

**Figure 1:**
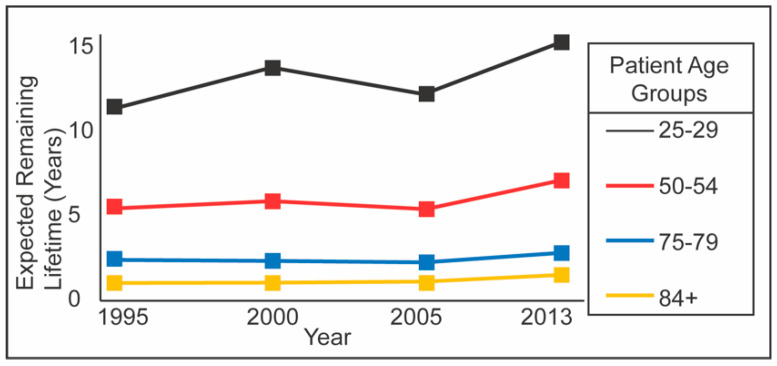
Expected remaining lifetime of end-stage renal disease (ESRD) patients on hemodialysis (HD) for the past two decades. For all age groups of ESRD patients, there has been no significant improvement in life expectancies [9]. This demonstrates a need for disruptive technologies in the field of hemodialysis.

While there have been many improvements in HD technology since Dr. Kolff constructed the first dialyzer in 1943 [6], only a few have occurred since the advent of the hollow fiber dialyzer. These improvements include high-flux membranes, automated safety mechanisms, dynamic monitoring of the rate of blood flow and hematocrit changes, and better volumetric ultrafiltration controls. However, the main HD methodology has remained the same since the 1970s [7]. Figure 1 shows the trend in expected remaining lifetimes for ESRD patients receiving HD as their renal replacement therapy (RRT) modality. Data was taken from the United States Renal Data System from the past two decades. The financial aspects are just as dismal. The total cost in the US alone exceeds $30 billion a year and $1 trillion worldwide [8]. Due to these limitations of current HD modalities, there arises a need for alternative methods, like continuous HD treatments.

Several types of ultrathin (≤ 100 nm) nanoporous, silicon-based membranes have been developed by our group and shown to improve efficiency and precision of size-based separations [5,10,11,12,13,14]. We reported recently a new type of nanoporous silicon nitride (NPN) membrane that is twice as strong as our original material and has the pore sizes appropriate for middle-weight serum toxin removal. Also reported were the results of single-pass benchtop studies with chip-formatted NPN (1.4 mm^2^) that demonstrate the extraordinary clearance potential of these membranes (10^5^ mL/min/m^2^), as well as their intrinsic hemocompatibility [5]. It is our hypothesis that these nanomembranes are able to reduce the form factor of HD delivery devices by orders of magnitude due to their thinness (100 to 1000 times thinner than commercial HD membranes). In previous work, we reported on in vitro studies that demonstrated the ability of nanomembranes to clear urea and middle-weight proteins while retaining albumin without degrading clearance rates over 12 h [15]. We have also tested 75 nm NPN in benchtop studies employing 5.4 mm × 5.4 mm chips with a single 0.7 mm × 2 mm membrane window [16]. Here, we describe the first examples of benchtop dialysis devices incorporating NPN sheet membranes and show that our single-pass, chip-based results are predictive of clearance in these larger area devices in recirculting studies. We have already shown that these NPN membranes (in a chip-based format) are able to reduce toxins in vivo in small-animal studies with 75 nm NPN membranes, in which urea levels were reduced by 26% in four hours of HD without reducing albumin detectably [5]. 

Hemocompatibility studies have been previously performed on the nanoporous nitride membranes [5]. These studies showed that the native NPN membranes have almost no propensity to precipitate immune activation (C3a generation) or coagulation (TAT generation) in ovine blood. Further, the ability of the NPN membranes to remove urea in small-animal hemodialysis for up to 4 h demonstrates the capability of the membranes to both clear uremic toxins and to resist fouling due to cellular adhesion.

Over the past decade, significant refinements have been made in the fabrication process, increasing our capacity to manufacture ultrathin membranes, maintaining a high yield while increasing the contiguous membrane area. The most dramatic example of this progress is the development of lift-off methods to create nanomembrane sheets with large active areas (up to 75 cm^2^) [17]. Successful incorporation of sheet membranes in devices compatible with small-animal hemodialysis experiments could achieve *Kt/V* ≥ 1.8 in the small-animal model in four hours, above the clinical target of *Kt/V* = 1.2. *Kt/V* is a measure of the HD dose commonly used to quantify hemodialysis treatment adequacy, where *K* = dialyzer urea clearance in mL/min, *t* = time in min, and *V* = the fluid volume of the patient in which urea is distributed.

Here, we present the fabrication of the NPN sheet membranes, which builds on the previously reported fabrication of chip-based NPN membranes [13] along with an analytical model for predicting the results of long-term continuous dialysis of a fluid volume from the results of short-term single-pass experiments. This model will enable the rapid development of fluidic systems to optimize for clearance while reducing (for example) the potential for hemolysis from excessive sheer forces.

## 2. Materials and Methods

### 2.1. NPN Membrane Fabrication 

Nanoporous nitride nanomembranes were fabricated following previously described processes [13], with modifications as follows. Thin films (silicon and silicon dioxide) were deposited on silicon nitride-coated wafers (50, 75 or 100 nm of silicon nitride). The silicon was converted to porous nanocrystalline silicon (32 nm or 40 nm) by a rapid thermal annealing step at 1050 °C for 5 min. (SSI Solaris 100 Rapid Thermal Processor) as previously described [10,18,19]. The pore size and density [18] were controlled by the thickness of the deposited thin films and other material properties along with the anneal parameters. Etching away the top oxide layer revealed the pnc-Si pore-transfer mask. An oxidation step made the porous nanocrystaline silicon (pnc-Si) thicker and more resistive as an etch mask by treatment at 1000 °C for 210 min in a tube furnace in an oxygen atmosphere (Bruce Technologies Inc., North Billerica, MA, USA). This porous silicon was converted to porous silicon oxide (oxidized mask) by this thermal oxidation step. The pores were transferred by a reactive ion etch (Trion RIE System, Trion Technology, Taiwan) to the underlying SiN. The oxidized mask was fully consumed in the transfer-etch process. 

### 2.2. Single-Pass Benchtop Dialysis with Chip-Based Devices

Single-pass benchtop studies were performed with chip-based devices, as described earlier [5]. Briefly, nanoporous membranes with supporting silicon chips, measuring 5.4 mm on each side, were used in benchtop dialysis experiments. Analyte fluid, phosphate-buffered saline (PBS) spiked with urea, flowed into a trench etched into the surface of the chip to come in contact with the membrane. Dialysate (analyte-free) fluid flowed on the opposite side of the membrane in a counter-flow manner. Fluid passed over the membrane only once and the analyte concentration entering the device was compared to that exiting, from which the analyte clearance in mL/min was calculated.

The area-normalized urea clearance rate, k*, was determined using the urea fractional loss,

(1) $$k^{*}=\left(f\cdot Q\right)/A_{m}$$

where ƒ is the fractional loss, *Q* is the analyte flow rate, and *A_m_* is the membrane area. Flow rates were increased in the same experimental setup and devices as in the previous experiment. Dialysate was always run at two times the analyte flow rate. The duration of the experiments was decreased as the flow rate increased in order to maintain a constant volume of fluid being pumped over the membrane for each flow rate. In order to determine whether reverse ultrafiltration was diluting the samples, fluid volumes were monitored.

### 2.3. Benchtop Dialysis Studies with NPN Sheet Membranes

Benchtop dialysis experiments used a blood surrogate solution comprised of 50 mg/dL urea (Millipore-Sigma, Burlington, MA, USA) in PBS. For all experiments, PBS pH 7.4 was used as the dialysate. For both blood and dialysate channels, a flow rate of 300 µL/min was used through the entire experiment with a set transmembrane pressure to achieve an ultrafiltration rate of 0.25 mL/h. Blood-side media was allowed to recirculate, while the PBS dialysate was run to endpoint over a four-hour dialysis duration. At specific timepoints, samples were removed for analysis to determine analyte concentration within the system. Urea concentrations were measured via a modified Jung reagent (OPA-Primaquine technique) method [20] using a urea assay standard as a reference (Millipore-Sigma, Burlington, MA, USA).

## 3. Results

### 3.1. Sheet Membrane Fabrication

**Figure 2:**
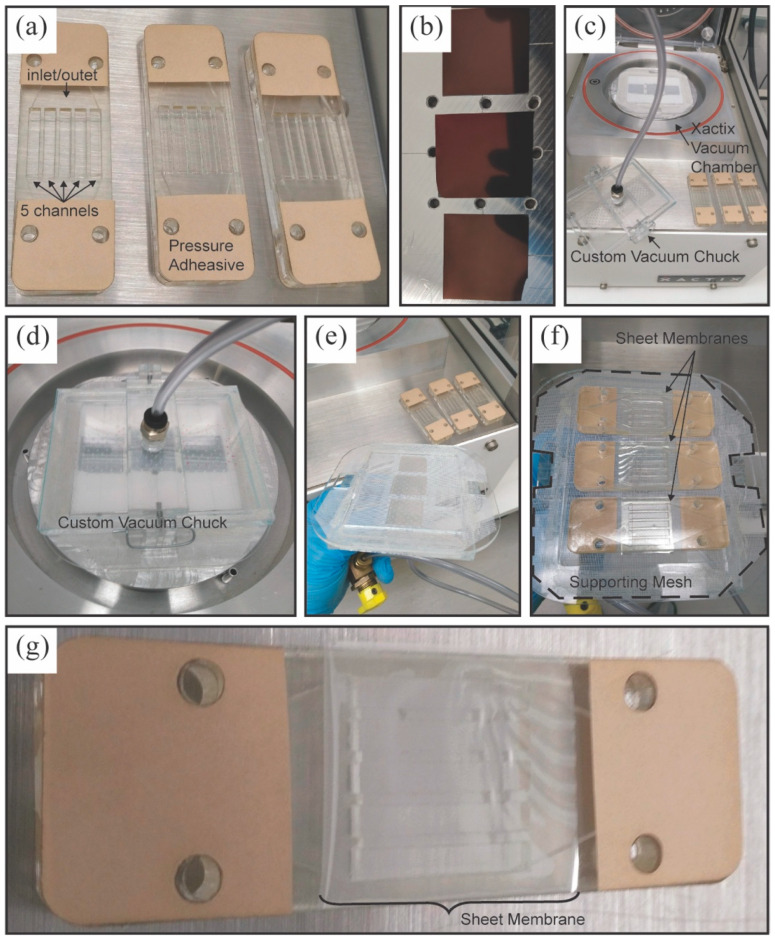
Sheet membrane fabrication process. (**a**) Assembled device halves ready for membrane transfer. (**b**) Loading three ~ 1″ × 1″ samples of nanoporous silicon nitride membrane wafers prior to release etch. (**c**) Etching using a Xactix^®^ E2 tool allows for transfer from the silicon wafer portions to the device frames. (**d**) Separating the mesh and sheet membranes from wafer samples. (**e**) Vacuum chuck inverted for transfer onto acrylic. (**f**) Devices applied to sheet membranes. (**g**) Device ready for leak check and sealing.

**Figure 3:**
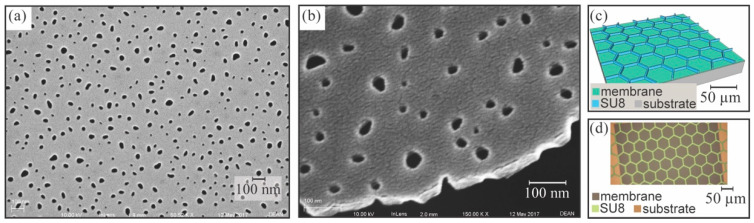
Nanoporous nitride oxide membrane. (**a**) SEM of nanoporous silicon nitride (NPN) membrane surface showing the pores. (**b**) SEM of cleaved NPN membrane showing relationship between pore size and membrane thickness. (**c**) Drawing of the patterned SU8 layer. (**d**) Optical image of the SU8 structure, here shown on a chip-based NPN membrane.

NPN sheet membranes were fabricated as above for releasing large areas of NPN membrane from the supporting wafer substrate Figure 2a–g. Figure 3a,b shows SEM images of the surface and edge of the NPN membrane. This process also required the patterning of a negative photo resist (SU8 3010; Microchem Corp., Westborough, MA, USA) support matrix onto the membrane to improve structural integrity during transfer to the dialyzer devices (see Figure 3c,d). The 3000-series SU8 was selected for its ability to be photo lithographically patterned. The 3000-series is robust, which is desirable for processing and membrane handling. It also has a relatively low film stress compared with earlier SU8 series [21]. This matrix allowed handling without membrane damage, at the expense of a 20% reduction in active membrane surface area. A mathematical model (based on Griffith’s law) that predicts burst pressures of thin nanoporous membranes was previously developed based on an effective fracture toughness [21]. The predicted burst pressure of the 500 kPa (72 psi) was based on the SU8 strut structure dimensions. This means that the strength of the membrane is limited to the strength of the SU8, which has yet to be tested.

A 10 µm SU8 layer patterned with 50 µm hexagonal openings (5 µm wide struts, ~80% open area) was used as a physical scaffold to reinforce the NPN membranes during the subsequent through-pore etch step using XeF_2_ (Xactix^®^ E2 Etcher, Allentown, PA, USA) that released the membranes from their wafer substrate. Under vacuum, the XeF_2_ diffused through the pores of the NPN to consume the poly-crystalline silicon proximally underlying the NPN layer, allowing for lift-off of the membrane by a custom vacuum transfer system. The thermal oxide layer acted as an etch stop for the XeF_2_ etchant.

### 3.2. Fluidic Device Assembly for NPN Sheet Membranes

**Figure 4:**
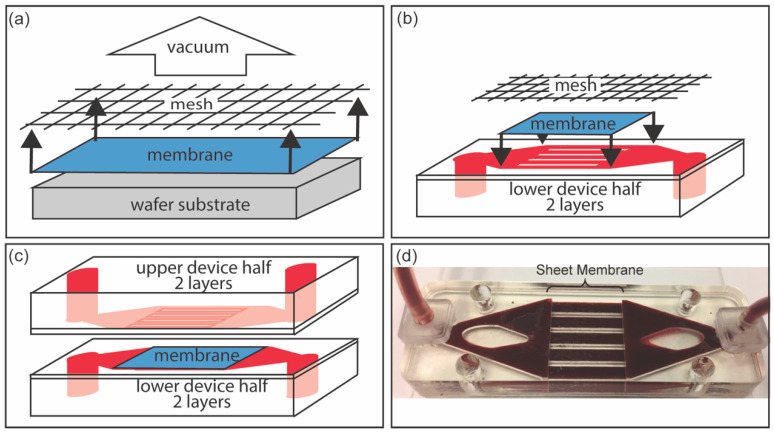
(**a**) Vacuum is applied through a mesh, to aid transfer, in order to lift the membrane from the wafer substrate after it is etched free in the Xactix^®^ E2. (**b**) The membrane is aligned with the bottom half of the device, which is made of two layers—one to support the membrane, and the other to hold the fluidic channels. (**c**) The upper half of the device is adhered to the lower half with pressure-sensitive adhesive. (**d**) The final device is leak tested and ready for clearance testing.

A simple device geometry was identified that enabled defect-free transfer of NPN sheet membranes from the Si support wafer to the prototype device—the stack geometry of which is shown in Figure 4. Importantly, this device yielded a significant increase (~200 times) in membrane surface area (288 mm^2^) relative to the chip-based devices (1.4 mm^2^) discussed earlier.

Identical halves of the fluidic device housing (see Figure 2a) were fabricated for membrane testing. Device layers for membrane interface (channel layer), fluid distribution, and device enclosure were laser cut from cast acrylic of either 1/16” or 1/4” thickness. Layers were assembled using a high-bond-strength double-sided pressure-sensitive adhesive (9474LE (300-LSE), 3M, Maplewood, MN, USA), and ports were installed to accept 1/16” ID tubing (5117K51, McMaster Carr Supply Company, Elmhurst, IL, USA). The NPN membrane was transferred from the silicon wafer after the lift-off etch to one half of the microfluidic device using a custom vacuum chuck as shown in Figure 2c. The membrane device was then examined for defects on the membrane surface via an optical inspection process which pressurized the device to 0.4 PSI of research grade Nitrogen gas. Any defects observed were repaired using liquid PDMS (Sylguard-184, Dow Corning, Midland, MI, USA) and tested for device function via verification of nitrogen fluency through the device at constant source pressure. The majority of devices had no defects, while some required up to three repairs. Final device assembly was completed by pressing the second half of the microfluidic device to activate the pressure sensitive adhesive.

### 3.3. Benchtop Dialysis Studies with NPN Sheet Membranes

**Figure 5:**
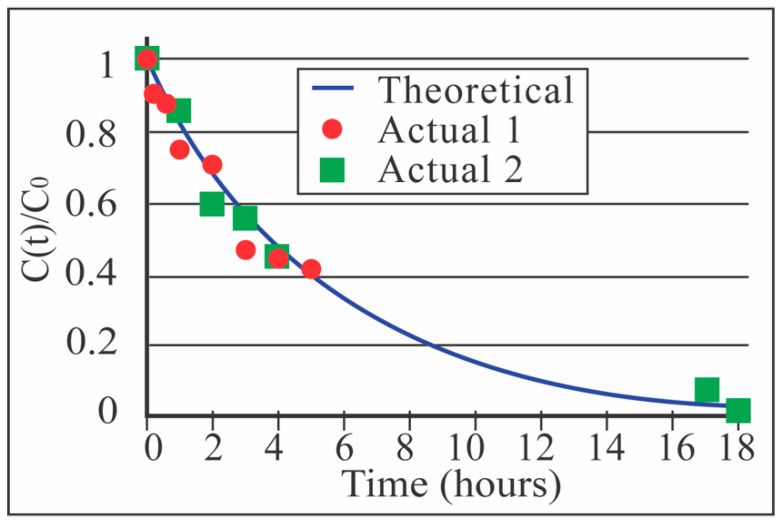
Comparison of the theoretical real-time urea clearance (see Equation (15)) to two multi-pass benchtop experiments with the sheet membrane device. Agreement was observed to match theory, thus showing that our model for $K=kr\left(A_{mp}/A_{sp}\right)kr\gamma$ is accurate (see Equation (18)). *A_mp_* = 2.88 × 10^–4^ m^2^; *A_sp_* =1.4 × 10^–6^ m^2^. (Experiment 1: R^2^ = 0.98; Experiment 2: R^2^ = 0.96.)

We created sheet membranes of 75 nm NPN material in order to increase the membrane area beyond what is easily achieved with chip-formatted membranes. We followed previously described methods [5,17], with some modifications. Briefly, NPN layers were fabricated as above on single side-polished, 375 µm thick silicon wafers, wherein the NPN layer was disposed on a 100 nm thick layer of poly-crystalline silicon (deposited by LPCVD) and a 100 nm thick, thermally grown silicon dioxide layer. A 10 µm thick SU8 layer (SU8 3010; Microchem Inc., Westborough, MA, USA) patterned with hexagonal openings (struts of 5 µm width, 50 µm hexagonal edge-to-edge width) and approximately 80% open area was used as a physical scaffold to reinforce the NPN membranes during the subsequent through-pore etch step using XeF_2_ (Xactix^®^ E2 Etcher, Allentown, PA, USA). Under vacuum, the XeF_2_ diffused through the pores of the NPN such that it consumed the poly-crystalline silicon proximally underlying the NPN layer, allowing for lift-off of the membrane by a custom vacuum transfer system. The thermal oxide layer acted as an etch stop for the XeF_2_ etchant. This lift-off technique was used to fabricate and release large areas of NPN membrane from the supporting wafer substrate (Figure 2a–c). This technique yielded a significant increase (~200 times) in membrane surface area (288 mm^2^) compared to the single-membrane chips described above (1.4 mm^2^). Device integrity was demonstrated via a leak check using suitable media (PBS stained with red food coloring). Further, the sheet membrane device was tested successfully using a variety of media including PBS, dilute serum, and whole blood (clearance results shown in Figure 5) at flow rates up to 3.5 mL/min without membrane or device failure, or hemolysis.

Unlike single-pass experiments with single-membrane chips, our sheet membrane studies recirculated the analyte solution (12.3 mL) through the device from a common media vessel to mimic the process used in the clinical setting. After four hours, the duration of a typical hemodialysis therapy, the average urea reduction ratio (URR) was 58.7% (n = 2; Figure 5). In terms of the more widely-used clinical parameter, the result was *Kt/V* = 0.88. While this is less than the clinical target of *Kt/V* = 1.2, either an increase in surface area from 288 mm^2^ to greater than 393 mm^2^ or an increase in flow rate can be implemented to achieve this target for an animal with a blood water volume of 12.3 mL (~190 g animal).

The urea concentration reduction over four hours was calculated with the starting urea concentration (*C*_0_ = 61.1 mg/dL) and the ending urea concentration (*C* = 25.3 mg/dL).

The urea reduction ratio (URR) was calculated.

(2) $$\mathrm{URR}=\left(1-\frac{C}{C_{0}}\right)\times 100$$

We calculated *Kt*/*V* (0.88) for these sheet membrane device experiments as follows:

(3) $$\frac{Kt}{V}=\ln\left(\frac{C_{0}}{C\left(t\right)}\right)$$

### 3.4. Analytical Model Relating Single-Pass Clearance, k, and Multi-Pass Clearance, K

In the context of dialysis, the ability of a membrane to remove toxins is represented as a clearance rate using Equation (2). It is important to note that dialysis systems are not setup in a single-pass fashion but are multi-pass systems, where the blood is continually circulated from the patients’ body, through the membranes, and back to the body. It is necessary to cast the urea clearance data into multi-pass terms for the data to be meaningful to the dialysis community. We start with the first-order rate equation to find the single-pass clearance,

(4) $$\frac{dC\left(t\right)}{dt}=-\kappa C\left(t\right)$$

where *κ* is the elimination rate constant for urea. This shows that the rate of urea loss is directly related to the concentration of urea above the membrane. Integrating Equation (4) yields,

(5) $$C_{e}=C_{0}e^{-\kappa t_{R}}$$

*t_R_* is the residency time for which fluid is in the dialyzer. The exit concentration from the dialyzer is proportional to the initial concentration before it is dialyzed.

Knowing *t_R_* is the dialyzed volume (*V_D_*) over the flow rate (*Q*),

(6) Ce=C0e−κVDQ

the elimination rate constant is defined as the fractional clearance per unit time (*κ* = *f*/*t*), which can be related to the simple-pass clearance (*k* = *f*∙*Q*), through the expression,

(7) $$∴\kappa =k/V_{D}$$

Using Equations (6) and (7),

(8) Ce=C0e−kQ

Now the main goal was to relate the single-pass to multi-pass to obtain a clear representation of how the membranes would perform in actual dialysis systems. For multi-pass, only a fraction of the sample is in the dialyzer at any given time, so a duty ratio is needed. The duty ratio, *r*, correlates the volume in the dialyzer to the total volume. Since the same flow rate is used throughout the entire system, *r* is also equal to the ratio of time in the dialyzer (*t_R_*) to the total circuit time (*t_C_*). The average fractional clearance in the multi-pass system will then be,

(9) $$\bar{k}=\frac{k\cdot t_{R}+0\cdot \left(t_{c}-t_{R}\right)}{t_{c}}=kr$$

We then substituted this into Equation (8),

(10) Ce=C0e−krQ

For the first loop through the device, where all fluid has passed by the dialyzer once,

(11) C1=C0e−krQ

We replaced *C_e_* with *C*_1_ to begin counting loops through the dialyzer,

(12) C2=C1e−krQ=C0e−2krQ

Generalizing for *n* loops,

(13) Cn=C0e−nkrQ

where

(14) n=tQV

(15) C(t)=C0e−krtV

We recalled the general equation for hemodialysis treatment,

(16) C(t)=C0e−KtV

where

(17) K=kr.

Equation (17) assume that the single-pass and multi-pass membranes are of equal size. If the surface areas are unequal, the value of *K* needs to be normalized for the area of the single-pass membrane (*A_sp_*) and denormalized for the area of the multi-pass membrane (*A_mp_*).

(18) $$∴\;K=kr\left(\frac{A_{mp}}{A_{sp}}\right)=kr\gamma$$

where *γ* = (*A_mp_/A_sp_*). With Equation (18), we have found a way to convert the quick and simple single-pass clearance studies to multi-pass clearance values that can be understood by the HD community.

The single-pass clearance, k* (area-normalized), approaches the mass transfer coefficient for the dialyzer *Ko* [22,23] as the analyte and dialysate flow rates are increased. With flow rate set to 4 mL/min in our dialyzer, *Ko* = k* = ~45,000 mL/min/m^2^, or ~7.5 × 10^2^ cm/s. Commercially available high-flux dialyzers, with a surface area of ~1.8 m^2^, have a *Ko* of ~560 mL/min/m^2^—smaller by two orders of magnitude than the *Ko* of our nanoporous membranes [24].

**Figure 6:**
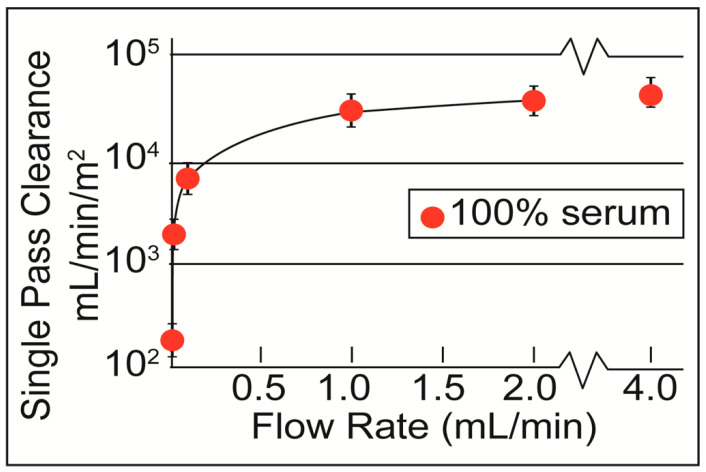
Single-pass clearance of urea in 100% serum, normalized to the membrane surface area. Adapted from Advanced Healthcare Materials, Wiley Publishing, 2020. [5].

From Figure 6, we estimate the normalized clearance, k*, for 300 mL/min in PBS (k* = 23,700 mL/min/m^2^). From k* and using *r* = 6.1 × 10^–2^ we calculate a normalized clearance. We calculated *r* from the ratio of *V_D_* to *V_t_*. Approximately one-third of the volume inside the dialyzer was actively being dialyzed (molecules in the upper 2/3 of the channel have no time to reach the membrane), and therefore *V_D_* was reduced to 0.075 mL. K* = 144.57 mL/min/m^2^ for the multi-pass experiments. We de-normalize with *A_mp_* = 2.88 × 10^–4^ m^2^ and find *K* = 0.042 mL/min. *C(t)/C*_0_ is calculated from *K*, *t* = 1080 min, and *V_t_* = 12.3 mL (the approximate blood volume of a Sprague–Dawley rat). We arrived at *Kt/V* = 0.82, and the calculated number from the multi-pass sheet membrane experiments was *Kt/V* = 0.88. The same relationship between single-pass and multi-pass clearance was plotted in Figure 5 using Equation (15) along with the experimental data and shows close agreement. (Actual 1: R^2^ = 0.98; Actual 2: R^2^ = 0.96.)

Having achieved clearance in both single-pass experiments with membrane chips (see Figure 6 [5]) and multi-pass experiments with sheet membranes (see Figure 5), we hypothesized that the simpler single-pass experiments were predictive of the multi-pass clearance, *K*. This will facilitate a much simpler development pathway for further membrane optimization on chips. By assuming that the analyte loss through the membrane is a steady first-order process, we derive K* = k*r (see Analytical Model in Methods), where r is a duty ratio describing the ratio of the volume being dialyzed, at any moment, to the total circuit volume in multi-pass experiments, and K* and k* are area-normalized clearance values for the multi-pass and single-pass systems, respectively. With this result, we predict that *C(t)/C*_0_ *= e^-krt/Vt^* for the multi-pass system, where *C(t)* is the analyte concentration, *C_0_* is the initial analyte concentration, *Vt* is the total volume of the multi-pass circuit and *t* is the dialysis time (see Analytical Model for values). Using the single-pass *k* values from Figure 6 evaluated at the flow rates in our multi-pass studies, we find excellent agreement between this prediction and actual data (Figure 5; Experiment 1: R^2^ = 0.98; Experiment 2: R^2^ = 0.96).

## 4. Discussion

Ultrathin HD membranes (nanomembranes) could represent one of the disruptive technologies needed for the revolutionary change in therapy that can improve longevity, lifestyle, and daily patient well-being. Here, we presented the fabrication of the NPN sheet membranes which built on the previously reported fabrication of chip-based NPN membranes [13] along with an analytical model for predicting the results of long-term continuous dialysis of a fluid volume from the results of short-term single-pass experiments. This model will enable the rapid development of fluidic systems to optimize for clearance while reducing the potential for hemolysis from excessive sheer forces.

Sheet membranes have been shown to have the ability to clear toxins at rates sufficient for small-animal dialysis. Future work will be undertaken to scale the devices for large-animal models and then clinical hemodialysis filters. The analytical equations presented here have been shown to make predictions about recirculating HD experiments from the results of single-pass experiments. Good results were shown from the blood surrogate used in this work. For future development of animal model studies, whole animal blood will need to be used in the single-pass study in order to account for the presence of cellular material.

Having a means to predict the clearance results of recirculating hemodialysis experiments with microdialyzers from the much easier to conduct single-pass experiments will enable a more rapid development of benchtop, animal model, and clinical hemodialysis devices. This is true of both standard fibrous membranes and our nanomembranes. The benefit of the sheet membrane material is the flexibility to incorporate it in many form factors for benchtop and small- to large-animal model devices as well as clinical hemodialysis filters.

## References

[1] USRDS (2019). United States Renal Data System. 2019 USRDS Annual Data Report: Epidemiology of Kidney Disease in the United States.

[2] Chien C.-W., Huang C.-J., Chao Z.-H., Huang S.-K., Chen P.-E., Tung T.-H. (2019). Hemodialysis interval and its association with emergency care and mortality. Medicine.

[3] Foley R.N., Gilbertson D.T., Murray T., Collins A.J. (2011). Long Interdialytic Interval and Mortality among Patients Receiving Hemodialysis. N. Engl. J. Med..

[4] Loutradis C., Sarafidis P.A., Papadopoulos C.E., Papagianni A., Zoccali C. (2018). The Ebb and Flow of Echocardiographic Cardiac Function Parameters in Relationship to Hemodialysis Treatment in Patients with ESRD. J. Am. Soc. Nephrol..

[5] Hill K., Walker S.N., Salminen A., Chung H.L., Li X., Ezzat B., Miller J.J., Desormeaux J.S., Zhang J., Hayden A. (2020). Second Generation Nanoporous Silicon Nitride Membranes for High Toxin Clearance and Small Format Hemodialysis. Adv. Health Mater..

[6] Kolff W.J. (1993). The beginning of the artificial kidney. Artif. Organs.

[7] Himmelfarb J., Ikizler T.A. (2010). Hemodialysis. N. Engl. J. Med..

[8] Ronco C., Davenport A., Gura V. (2008). A wearable artificial kidney: Dream or reality?. Nat. Clin. Pract. Nephrol..

[9] USRDS (2018). United States Renal Data System. 2018 USRDS Annual Data Report: Epidemiology of Kidney Disease in the United States.

[10] Striemer C.C., Gaborski T.R., McGrath J.L., Fauchet P.M. (2007). Charge- and size-based separation of macromolecules using ultrathin silicon membranes. Nature.

[11] Snyder J., Clark A., Fang D., Gaborski T., Striemer C., Fauchet P., McGrath J.L. (2011). An experimental and theoretical analysis of molecular separations by diffusion through ultrathin nanoporous membranes. J. Membr. Sci..

[12] Gaborski T.R., Snyder J.L., Striemer C.C., Fang D.Z., Hoffman M., Fauchet P.M., McGrath J.L. (2010). High-Performance Separation of Nanoparticles with Ultrathin Porous Nanocrystalline Silicon Membranes. ACS Nano.

[13] Desormeaux J.P.S., Winans J.D., Wayson S.E., Gaborski T.R., Khire T.S., Striemer C.C., McGrath J.L. (2014). Nanoporous silicon nitride membranes fabricated from porous nanocrystalline silicon templates. Nanoscale.

[14] Smith K.J.P., Winans J., McGrath J.L. Ultrathin Membrane Fouling Mechanism Transitions in Dead-End Filtration of Protein. Proceedings of the ASME 2016 14th International Conference on Nanochannels, Microchannels, and Minichannels.

[15] Johnson D.G., Khire T.S., Lyubarskaya Y.L., Smith K.J., Desormeaux J.-P.S., Taylor J.G., Gaborski T.R., Shestopalov A.A., Striemer C.C., McGrath J.L. (2013). Ultrathin Silicon Membranes for Wearable Dialysis. Adv. Chronic Kidney Dis..

[16] Mossu A., Rosito M., Khire T., Chung H.L., Nishihara H., Gruber I., Luke E., Dehouck L., Sallusto F., Gosselet F. (2018). A silicon nanomembrane platform for the visualization of immune cell trafficking across the human blood–brain barrier under flow. Br. J. Pharmacol..

[17] Miller J.J., Carter R.N., McNabb K.B., Desormeaux J.-P.S., Striemer C.C., Winans J.D., Gaborski T.R. (2014). Lift-off of large-scale ultrathin nanomembranes. J. Micromech. Microeng..

[18] Fang D.Z., Striemer C.C., Gaborski T.R., McGrath J.L., Fauchet P.M. (2010). Methods for controlling the pore properties of ultra-thin nanocrystalline silicon membranes. J. Phys. Condens. Matter.

[19] Qi C., Striemer C.C., Gaborski T.R., McGrath J.L., Fauchet P.M. (2014). Highly Porous Silicon Membranes Fabricated from Silicon Nitride/Silicon Stacks. Small.

[20] Zawada R.J., Kwan P., Olszewski K.L., Llinás M., Huang S.-G. (2009). Quantitative determination of urea concentrations in cell culture medium. Biochem. Cell Biol..

[21] Gillmer S.R., Fang D.Z., Wayson S.E., Winans J.D., Abdolrahim N., Desormeaux J.-P.S., Getpreecharsawas J., Ellis J., Fauchet P.M., McGrath J.L. (2017). Predicting the failure of ultrathin porous membranes in bulge tests. Thin Solid Films.

[22] Ouseph R., Ward R.A. (2001). Increasing Dialysate Flow Rate Increases Dialyzer Urea Mass Transfer-Area Coefficients During Clinical Use. Am. J. Kidney Dis..

[23] Leypoldt J.K., Cheung A.K., Agodoa L.Y., Daugirdas J.T., Greene T., Keshaviah P.R., Beck G.J. (1997). Hemodialyzer mass transfer-area coefficients for urea increase at high dialysate flow rates. Kidney Int..

[24] Hootkins R. (2011). Lessons in dialysis, dialyzers, and dialysate. Dial. Transplant..

